# Disentangling the perceptual underpinnings of autism: Evidence from a face aftereffects experiment

**DOI:** 10.1002/aur.3283

**Published:** 2024-12-16

**Authors:** Julius Hennig, Arne Doose, Clara Marie Breier, Alexander Soutschek, Nicole Beyer, Stefan Schweinberger, Ingeborg Kamp‐Becker, Luise Poustka, Katja Albertowski, Veit Roessner, Stefan Ehrlich

**Affiliations:** ^1^ Translational Developmental Neuroscience Section, Division of Psychological and Social Medicine and Developmental Neurosciences, Faculty of Medicine Dresden TU Germany; ^2^ Department of Psychology Ludwig Maximilian University Munich Munich Germany; ^3^ Department of Child and Adolescent Psychiatry and Psychotherapy, Medical Faculty Technische Universität Dresden Dresden Germany; ^4^ Department of General Psychology and Cognitive Neuroscience Friedrich Schiller University Jena Germany; ^5^ German Center for Mental Health (DZPG), Site Jena‐Magdeburg‐Halle Jena Germany; ^6^ Social Potential in Autism Research Unit Friedrich Schiller University Jena Germany; ^7^ Department of Child and Adolescent Psychiatry, Psychosomatics and Psychotherapy, Faculty of Human Medicine Philipps‐University Marburg Marburg Germany; ^8^ Department of Child and Adolescent Psychiatry, Centre for Psychosocial Medicine University of Heidelberg Heidelberg Germany

**Keywords:** autism, face aftereffects, gender processing, hierarchical drift diffusion modeling, perceptual processing

## Abstract

Existing literature has documented diminished norm‐based adaptation (aftereffects) across several perceptual domains in autism. However, the exact underlying mechanisms, such as sensory dominance possibly caused by imprecise priors and/or increased sensory precision, remain elusive. The “Bayesian brain” framework offers refined methods to investigate these mechanisms. This study utilized both model‐free (frequentist statistics) and model‐based (hierarchical Drift Diffusion Modeling) analytical approaches to compare gender face aftereffects in male adolescents with autism (*n* = 29) to neurotypical controls (*n* = 39) using a behavioral choice experiment. Contrary to our initial hypotheses, our analyses did not find support for imprecise priors or increased sensory precision within the autistic group. Instead, we observed generally decreased drift rates towards male but not female stimuli in the autistic group. Thus, our findings suggest a lack of own‐gender bias in face processing among the autistic participants. These findings align with more recent behavioral and neurophysiological research observing intact priors in individuals with autism, suggesting that other mechanisms may better explain the perceptual challenges in autism. Our study contributes to the ongoing discourse on perceptual processing in autism, emphasizing the necessity for more nuanced analytical approaches in order to unravel the complexity of this condition.

## Lay Summary

Our study investigated face gender recognition in male adolescents with autism compared to their healthy male peers, using advanced statistical models. Contrary to what we expected, our findings showed that individuals with autism processed gender information in faces similarly to healthy young men. The results indicate that these basic perceptual abilities might be intact. This suggests that the common theory that autism involves faulty basic perceptual processes might need revisiting, offering a new perspective on how perception works in autism.

## INTRODUCTION

Autism spectrum disorder (ASD) is a neurodevelopmental disorder characterized by persistent deficits in social communication and social interaction, accompanied by restricted, repetitive patterns of behavior, interests, or activities (Lord et al., 2020). These difficulties can range from mild to severe, affecting a person's ability to function in daily life. Initial theories identified social deficits as the primary symptoms in ASD (Baron‐Cohen, 1989; Baron‐Cohen et al., 1985; Chevallier et al., 2012). Later research suggests that these social deficits are secondary, possibly emerging from perceptual deficits (Happé & Frith, 2006; Mottron et al., 2006). However, the underlying neural mechanisms remain unclear.

**FIGURE 1:**
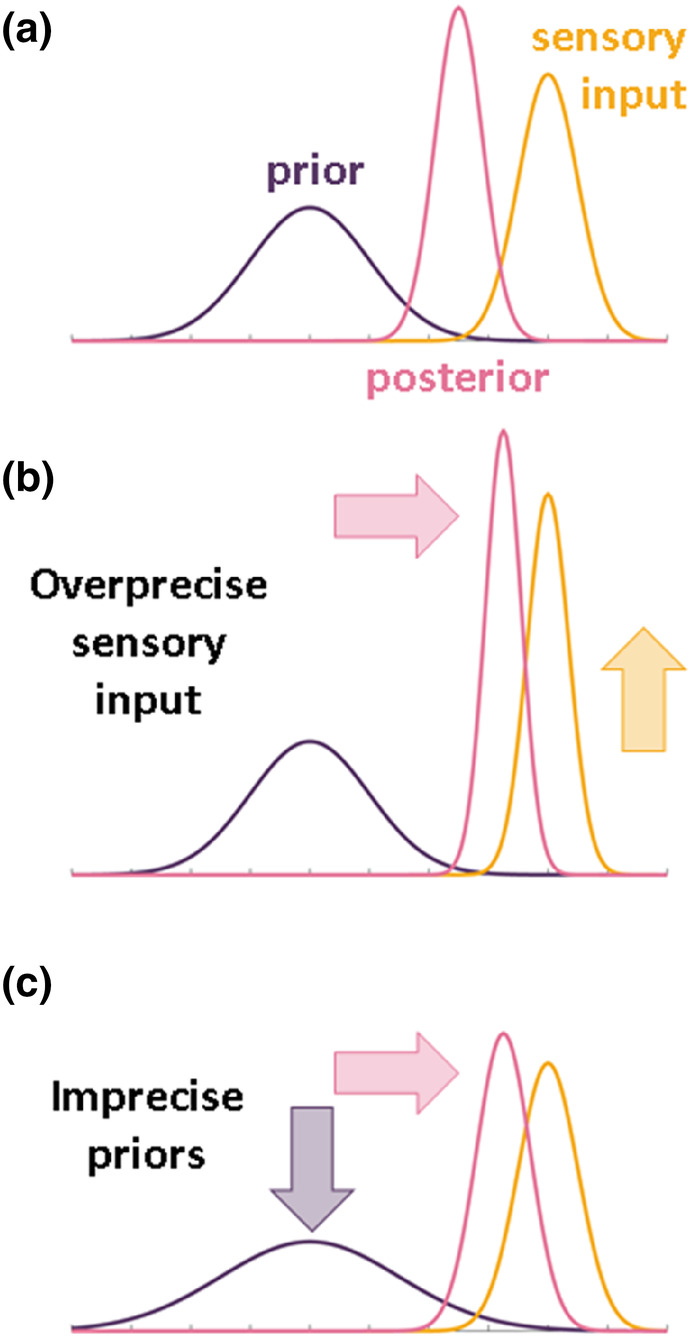
(a) Illustration of Bayesian perception of the world, where the prior (existing belief) and the bottom‐up sensory input are optimally combined using Bayes' rule, yielding a posterior (resultant perception) that represents updated beliefs after considering the new evidence. (b) Shows the effect of increased sensory precision, where a narrower and taller distribution signifies more precise sensory information (yellow arrow). This precision enhances the influence of sensory input on the perception (pink arrow). (c) Depicts the scenario of imprecise priors, characterized by a wider and flatter prior distribution (purple arrow), indicating less confidence in initial beliefs. This imprecision dilutes the prior's influence, resulting in a posterior that shifts towards the sensory input distribution. Both (b) and (c) lead to a sensory dominance during perception.

More recent theoretical frameworks, such as predictive coding, offer testable hypotheses explaining altered perception in individuals with autism and its association with other symptoms (Haker et al., 2016; Pellicano & Burr, 2012; Van De Cruys et al., 2014). These “Bayesian brain” models conceptualize perception as an inferential process based on a priori beliefs and sensory information, in which both are weighted by their assumed precision (Friston, 2009; Petzschner et al., 2017). In regard to autism, it is hypothesized that a shift in precision weights leads to sensory dominance over prior beliefs. However, the specific mechanisms of this sensory dominance remain unclear, with both heightened sensory precision (Lawson et al., 2014; Palmer et al., 2017; Van De Cruys et al., 2013) and imprecise priors (Pellicano & Burr, 2012) being potential explanatory factors of the observed perceptual challenges (Figure 1; for an illustrative example of Bayesian models of perception, see Supporting Information: 1). This in turn can lead to overfitting and a lack of generalization in autism, characterized by focusing too narrowly on specific sensory details at the expense of broader context or patterns; such a focus makes it hard to apply knowledge to new, slightly different situations (Schneebeli et al., 2022), which may provide an explanation for the varied clinical symptoms described above, especially the social communication problems in highly unpredictable, volatile environments, for example, in novel situations or in social interactions.

Building upon the theoretical background provided by the aforementioned models in understanding the perceptual intricacies of autism, the Bayesian hierarchical drift diffusion model (hDDM) allows disentangling the complex processes of perception in decision‐making. In general, DDMs conceptualize decision‐making as a continuous process, where evidence accumulates over time until a threshold is reached, resulting in a decision (Ratcliff et al., 2016; Ratcliff & McKoon, 2008). This approach is particularly adept at delineating the temporal dynamics of decision‐making, distinguishing between the speed of information accumulation, the amount of information required to make a decision as well as biases towards certain decisions. In the context of autism, the presence of increased sensory precision could manifest in an accelerated rate of information accumulation. Additionally, imprecise priors in autism could be reflected both in the starting point and the rate of information accumulation, that is, these parameters may be less influenced by prior beliefs, especially when these differ from new sensory input.

The potential of hDDM as a Bayesian analytical approach becomes apparent when considering face processing, a crucial component of social communication and interaction, in the context of autism. In general, studies have shown that individuals with autism experience challenges in processing and identifying faces and recognizing emotions from facial expressions (Tang et al., 2015; Webb et al., 2017a; Weigelt et al., 2012). More specifically, they tend to exhibit reduced accuracy and prolonged reaction times compared to neurotypical (NT) individuals, particularly in tasks involving emotion and gaze processing. However, most studies so far used model‐free analytical approaches, so it remains largely unclear how exactly face processing may be altered. Accordingly, (Tang et al., 2015) concluded in their review that individuals on the autism spectrum showed differences in face processing compared to NT individuals both quantitatively (“how much”) and qualitatively (“how”). One approach to investigate such qualitative alterations in autism is the norm‐based “face space.” This theoretical framework posits a multidimensional space in which faces are stored along dimensions that capture characteristics that can vary independently (Trapp et al., 2018; Valentine et al., 2016; Wuttke & Schweinberger, 2019). Typically, in order to build a perceptual norm (i.e., the center of this face space), the “average” face is continuously updated with new face‐related sensory input (Rhodes, 2017). Experimentally, this concept can be explored through the face aftereffect (FAE), a perceptual phenomenon in which exposure to a face or a particular facial feature, such as gender or gaze direction, can temporarily alter the perception of subsequent faces. Essentially, prolonged exposure to a face or a facial feature can cause the visual system to adapt to that stimulus, resulting in a biased perception of subsequently viewed faces towards the opposite on the dimension of the relevant facial feature (Webster & MacLeod, 2011). This means that the phenomenon of FAE allows to manipulate the norm face (Webster & MacLeod, 2011) by moving the norm towards the adaptor stimulus (thus changing the prior). In autistic children and adolescents, several studies have documented reduced aftereffects during face processing (Ewing et al., 2013; Pellicano et al., 2007; Rhodes et al., 2014), indicating that atypical adaptive norm‐based face coding may contribute to difficulties in face processing in autism. Furthermore, (Pellicano et al., 2013) and (Rhodes et al., 2018) reported reduced gaze direction and facial expression aftereffects, respectively, in children on the autism spectrum. Interestingly, there is also some evidence that these challenges in norm‐based face coding may diminish when transitioning to adulthood (Walsh, Maurer, et al., 2015; Walsh, Vida, et al., 2015). A notably understudied area within the context of the FAE in autism research is the gender FAE, with no published studies as of yet. While analyses of figural aftereffects to spatially distorted faces provided evidence for the existence of partially dissociable male and female facial prototypes that are similar for adults with ASD and NT adults (Walsh, Vida, et al., 2015), evidence regarding aftereffects that are directly induced by variations in facial gender is lacking. This seems important especially since gender typicality in faces might be a possibly relevant factor in reported gender classification difficulties in individuals with ASD in multiple age groups (Strauss et al., 2012). However, given the impact it has on social interaction and communication for example, gender classification seems to confound emotion perception from facial cues (Adams et al., 2015), gender recognition skills are crucial and difficulties in it may have a considerable impact in daily life, especially as face processing abilities might be related to the development of social skills (Webb et al., 2017b).

The aim of this study was to investigate the behavioral basis of the gender FAE in participants on the autism spectrum compared to NT individuals, employing an established experimental paradigm (Kloth et al., 2010). As a first step, we utilized a model‐free analysis approach, leveraging frequentist statistics to explore basic behavioral alterations in face adaptation. Next, we adopted a model‐based analysis approach utilizing hDDM analysis with its capabilities to capture individual differences in information accumulation speed, decision thresholds and starting points (biases) systematically across the autism and NT groups. Considering the hypothesis of potentially increased “sensory precision” in autism, we expected that participants with autism would exhibit an increased rate of information accumulation in the hDDM model. In relation to “imprecise priors” in autism, our expectation was a less pronounced FAE on behavioral metrics such as information accumulation speed and the starting point.

## METHODS

### Sample description

This study was part of the ASD‐net study (Kamp‐Becker et al., 2017). For this experiment, we recruited 33 male participants on the autism spectrum. Of those, data of 29 participants were used in this study (*n* = 2 did not complete the experiment; *n* = 2 did not respond in >33% of the trials). Independently of the ASD‐net study, we also recruited 43 male NT individuals through advertisement among middle school and high school students as control participants. Of those, 39 were included in this study (*n* = 2 did not complete the experiment, n = 2 did not respond in >33% trials). Taken together, data of 68 individuals was used in this study. Participant demographics as well as clinical characteristics are provided in Table 1.

**TABLE 1:** Demographic and clinical characteristics of the sample.

	AUT			NT				
	*n*	*M*	SD	*n*	*M*	SD	*t*	*p*
Age	29	14.83	1.95	39	15.03	1.87	0.42	0.673
IQ	20	109.2	11.76	33	112.3	8.58	1.06	0.296
SRS (parents)	23	80.39	8.13	39	41.74	10.34	15.3	<0.001
DIKJ	23	30.23	7.2	32	6.59	3.85	11.94	<0.001
AQ	‐	‐	‐	37	12.30	4.55	‐	‐
SCL‐90‐R GSI	‐	‐	‐	35	44.37	8.53	‐	‐

*Note*: Due to technical difficulties, several questionnaire data were lost. In the AUT sample, *n* = 6 reported intake of psychotropic medication and *n* = 3 were diagnosed with comorbid psychiatric disorders; Supporting Information: 2 for more details.

Abbreviations: AUT, individuals on the autism spectrum; AQ, autism quotient; DIKJ, depression inventory for children and adolescents; NT, neurotypical individuals; SCL‐90‐R GSI, Symptom Checklist‐90 Revised Global Severity Index; SRS, Social Responsiveness Scale.

This study was approved by the Institutional Review Board of the Technische Universität Dresden and carried out in accordance with the latest version of the Declaration of Helsinki, and all participants (and their guardians if underage) gave written informed consent.

Autistic individuals were included when they were diagnosed with either childhood autism (F84.0), Asperger syndrome (F84.5) or atypical autism (F84.1) according to the ICD‐10 criteria. NT individuals were included when they did not show any indications of ASD symptomatology and no other psychiatric symptoms. General inclusion criteria were: age 12 ≤ years ≤ 18; IQ > 75; no or stable psychotropic medication (stable medication for 4 weeks prior to randomization); participants and parents/caregivers had to be fluent in German; and written informed consent by legal guardian of the participants, and assent of the participants themselves. For more details regarding exclusion criteria, see the Supporting Information: 2.

### Measures

To ascertain the diagnosis in our sample on the autism spectrum, several assessments commonly used in both research and clinical settings were conducted by trained clinical psychologists or physicians in an outpatient clinic specialized in autism: the ADOS‐2, a semi‐structured, standardized assessment that involves direct observation of an individual's social interaction, communication, and play behaviors (Poustka et al., 2015), the ADI‐R, a structured, standardized interview that is administered to a caregiver or family member of the individual being evaluated for autism (Bölte et al., 2006), and the Social Responsiveness Scale for parents (SRS; Constantino et al., 2003). To confirm the absence of ASD symptoms in the NT, we used the SRS and the autism‐spectrum quotient (AQ), a self‐report questionnaire designed to measure traits associated with autism in individuals without a clinical diagnosis of ASD (Baron‐Cohen et al., 2006). These measures were conducted by clinically experienced and trained research assistants under the supervision of the attending child and adolescent psychiatrist.

To ascertain that the NT participants had no other psychiatric disorders, we conducted the MINI‐KID (Lecrubier et al., 2010) and SCL‐90‐R (Franke & Derogatis, 2002) regarding general psychopathology. To assess symptoms of depression, we used the “Depressions‐Inventar für Kinder und Jugendliche” (DIKJ; Depression Inventory for Children and Adolescents), a German‐language self‐report questionnaire (Stiensmeier‐Pelster et al., 2000). For more details regarding the diagnostic procedure and instruments, see Supporting Information: 3.

### Stimuli

**FIGURE 2:**
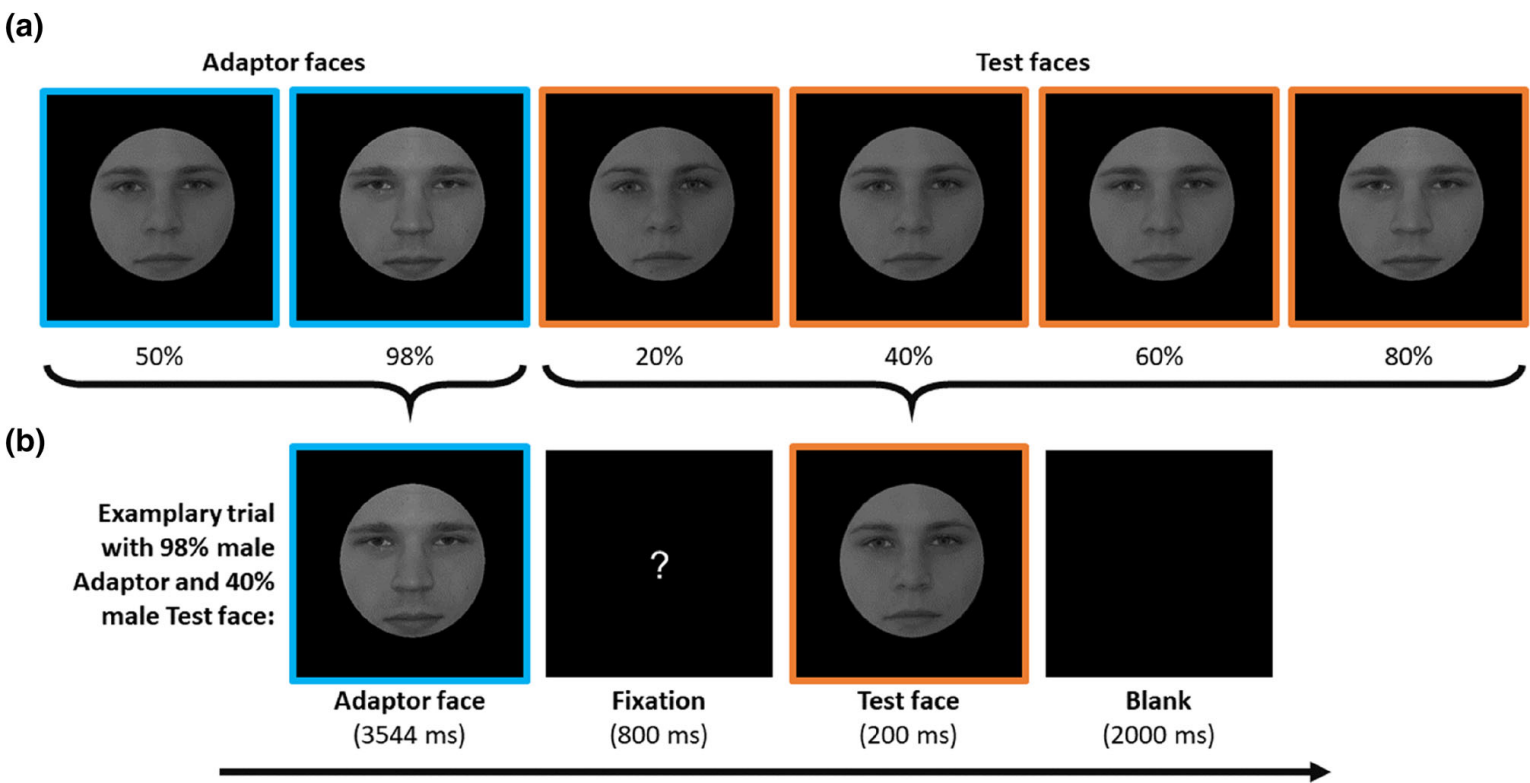
(a) Examples of stimuli of one male–female pairing. Orange‐bordered stimuli are Test face stimuli (20%, 40%, 60%, 80% male) and blue‐bordered are Adaptor face stimuli (androgynous: 50% male; male: 98% male). These colored borders are only for visualization purpose and were not used in the experiment. (b) Exemplary trial procedure with a male Adaptor and a 40% male test stimulus.

The face stimuli used in this study were the same as in the study of (Kloth et al., 2010). They were grayscale full‐front digital photographs of four young men and four young women and free of features that are typically perceived as gender‐specific, such as hair and make‐up. The faces were fitted behind an oval mask to hide the outer contours and morphed on a male–female axis (for more details regarding the morph process, see (Kloth et al., 2010) and (Kovács et al., 2006)). Ultimately, we used four androgynous face images (50%:50% male to female ratio) and four male face images (98%:2% male: female ratio) as androgynous and male adaptor images. As test stimuli, we used 16 images of four faces with different Morph levels (20%:80%, 40%:60%, 60%:40%, and 80%:20% male: female ratio). All face stimuli measured 6.5 × 6.5 cm and were presented on a black screen (Figure 2a).

### Experimental procedure

Participants in this study went through two face adaptation conditions based on the experimental setup in the study by (Kloth et al., 2010). These conditions either contained ambiguous gender information (androgynous adaptation) or unambiguous gender information (male adaptation, see Figure 2b). The order of gender of adaptation stimuli was counterbalanced across participants, and they were tested in separate blocks. Each condition consisted of 32 repetitions of each of the four Morph levels, resulting in a total of 128 trials per condition. In each trial, participants were first presented with the adaptor for 3544 ms, followed by a white question mark on a black screen for 800 ms, indicating that participants had to respond to the following test face, which was presented for 200 ms. After that, a black screen was shown for 2000 ms, during which participants responded. Participants were instructed to determine the gender of the test faces and classify them as either male or female by pressing one of two marked keys labeled “M” and “F” on a standard keyboard. This experiment was conducted on a laptop (Fujitsu Lifebook A530).

### Statistical analyses

We analyzed data with model‐free and model‐based analyses. Following (Kloth et al., 2010) for the model‐free analyses, “male” responses were analyzed using a 2 × 2 × 4 mixed ANOVA with Adaptor gender and Morph level (of test stimulus) as within‐subject factors and Group as a between‐subject factor. We tested for homogeneity using Levene's test, and for sphericity using Mauchly's test. Since the Mauchly's test was significant, indicating a violation of sphericity, we applied the Greenhouse–Geisser correction. These statistical analyses were performed using SPSS (IBM SPSS Statistics 28.0.0.1). The differences between the androgynous and male Adaptor conditions are considered as the “FAE.”

To test whether the autistic participants' altered behavior in the FAE task can be explained by changes in how they accumulate evidence, we computed hierarchical drift diffusion models (hDDM; Johnson et al., 2017) which allows embedding DDMs in a hierarchical framework and to estimate it using Bayesian methods. One of the benefits of hDDM over classical DDM is the estimation of parameters at individual and condition levels simultaneously based on variable group level distributions, reducing the influence of individual outliers and making it possible to work with a smaller amount of data per participant. To ensure data quality, we excluded the first three trials, response omission trials (similar to studies such as Donzallaz et al., 2023), and trials with extremely fast (<250 ms) or slow (>3 SD above the mean: >1640 ms) reaction times. In addition, we excluded 2 NT participants for the hDDM who had less than 50% valid trials remaining after applying these criteria, resulting in a total for *n* = 29 autistic and *n* = 37 NT participants. We estimated the following DDM parameters: drift rate *v* (speed of information accumulation), starting point *b* (initial bias in favor of one option), boundary *a* (amount of information needed to make a decision), and non‐decision time *t* (processes not involved in decision‐making, such as stimulus encoding and motor response). All parameters were estimated separately for each group. Additionally, drift rates *v* were calculated for each Adaptor and Morph level, and the starting point *b* for each Adaptor. In summary, we estimated the posterior distributions for a total of 24 parameters.

For our analysis, we modeled the likelihood of making the binary decision within a specific reaction time using the Wiener first passage time (wfpt) distribution:

$${\mathrm{rt}}_{o,i}\sim \text{wfpt}\left(t_{p},a_{p},b_{p,a},v_{p,a,m}\right),$$

where rt is the response time (RT) for choosing option *o* on trial *i* (positive RTs indicating a male response and negative RTs indicating a female response). The wfpt calculates the probability of choosing *o* with the response time rt based on the four parameters. For the hierarchically structured model, priors were sampled from normal distributions for each participant *p* using specific means μ and precision τ (inverse of the variance) of the relevant higher order condition‐levels (for group *g*, adaptor *a* and Morph level *m*):

$$a_{p}\sim N\left(\mu_{g}^{a},\tau_{g}^{a}\right),$$

$$t_{p}\sim N\left(\mu_{g}^{t},\tau_{g}^{t}\right),$$

$$b_{p}\sim N\left(\mu_{g,a}^{b},\tau_{g,a}^{b}\right),$$

$$v_{p}\sim N\left(\mu_{g,a,m}^{v},\tau_{g,a,m}^{v}\right).$$

We ran the model in the Markov Chain Monte Carlo (MCMC) sampler JAGS (Version: 4.3.0; Plummer, 2003) with the Wiener module extension (Wabersich & Vandekerckhove, 2014) and used R (4.2.0; R Core Team, 2023) as well as the R package “R2jags” (Version: 0.7–1).

We estimated the posterior distributions of our parameters by computing two independent Markov chains with 20,000 iterations each, with the first 15,000 iterations discarded as burn‐in samples. Convergence of the remaining 5000 samples was assessed using visual inspection of the MCMC trace plots and $\hat{R}$ values (with all posterior distributions having $\hat{R}$ values below 1.01, Supporting Information: 4). For each parameter, we described the posterior distribution by their median value and a high density interval (HDI).

For hypothesis testing, we calculated the differences between the posterior distributions for two parameters by subtracting their HDIs. More specifically, for each contrast of interest, we computed 5000 differences based on the 5000 iterations of each parameter estimation. For example, when testing group differences, we subtracted the values based on 5000 iterations of the autism group from the 5000 values of the NT group. For more complex effects and interactions, we computed nested subtractions (an example is provided in Supporting Information: 5). To control for false positives, we applied the false discovery rate (FDR) correction as proposed by (Benjamini & Hochberg, 1995) with a significance level of *q* = 0.05. The resulting corrected significance threshold was used to determine the HDI distribution boundaries. If the resulting HDI distribution of mean differences did not contain zero, the condition effect was considered credible and thus significant (Johnson et al., 2017). In this study, the FDR‐adjusted significance threshold was *p*_FDR‐adjusted_ = 0.027. Accordingly, the HDI interval was set to 97.3% for testing HDI differences with boundaries at 1.35% and 98.65%.

Finally, we performed posterior predictive checks (PPC) in order to validate the hDDM parameter estimates. To this end, we used the posterior distribution of the model parameters and generated multiple sets of data by simulating response times and choices for each trial and subject as well as all experimental conditions. We then used visual plots to assess the goodness‐of‐fit and thus our model's validity.

## RESULTS

### Sample characteristics

There were no significant age or IQ differences between the autism and the NT group (Table 1). As expected, the autism group had significantly higher SRS and depression (DIKJ) scores compared to the NT group. Importantly, the NT group reported no symptoms or showed no behaviors suggestive of autistic traits according to the AQ, validating our recruitment strategy. Additionally, the NT group did not show any psychiatric symptoms as obtained by the SCL‐90‐R.

### Model‐free analysis of the FAE

**FIGURE 3:**
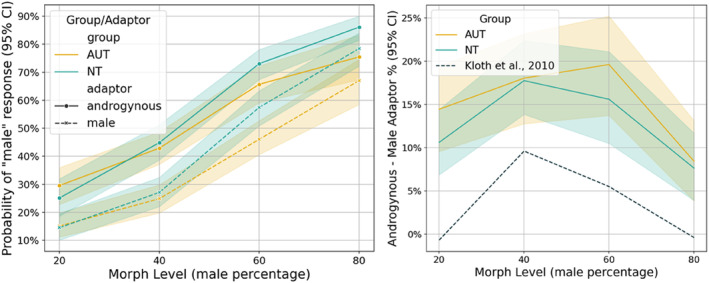
Line diagrams of the percentage means (and 95% confidence interval; CI) of the face aftereffect (FAE) experiment. Left: Probability of male responses for each Morph level and separated for both groups and for both adaptor conditions. Right: FAE depicted by the difference in male response probabilities between the androgynous and the male Adaptor, separated for both groups. Additionally, with the black dashed line, we included the results in the study by Kloth et al. (2010) with adult participants as reference. The inverted U‐shaped curve is indicative of the FAE as the uncertainty is higher in the more ambiguous 40% and 60% Morph levels. AUT, individuals on the autism spectrum; NT, neurotypical individuals.

We first compared the FAE between the autism and NT groups with conventional model‐free analyses of decisions in the FAE task. A 2 × 2 × 4 ANOVA (Greenhouse‐Geisser corrected) was conducted, with Adaptor gender and Morph level as within‐subject factors and Group as a between‐subject factor. The analysis revealed significant main effects of Morph level (*F*(1.47, 97.05) = 247.95, *p* < 0.001) and Adaptor (*F*(1, 66) = 158.52, *p* < 0.001), confirming that the task worked as intended. There was no main effect of Group (*F*(1, 66) = 2.70, *p* = 0.105), but a significant Morph level × Group interaction (*F*(1.47, 97.05) = 3.96, *p* = 0.034), indicating that autistic individuals showed difficulty accurately identifying the test faces (relative to NT), particularly for the less ambiguous morph levels (20% and 80% male). Additionally, the Morph level × Adaptor interaction was significant (*F*(2.29, 151.73) = 8.56, *p* < 0.001), indicating that the FAE leads to a stronger decrease in the likelihood of choosing the male response. The Adaptor × Group (*F*(1, 66) = 1.01, *p* = 0.320) and Morph level × Adaptor × Group interactions (*F*(2.29, 151.37) = 0.38, *p* = 0.716) did not reach significance (Figure 3; see Supporting Information: 6 for post‐hoc tests and a Bayesian 2 × 2 × 4 ANOVA). Taken together, using classical frequentist analysis, the autistic group demonstrated intact face adaptation while showing general difficulties in gender‐specific face classification. With respect to the aim of this study, these findings based solely on choice data suggest intact priors contrary to the hypothesis of inflexible priors. However, while the frequentist analysis has provided initial insights, it does not fully elucidate the underlying cognitive processes and decision‐making dynamics. Therefore, we employed model‐based hDDM analysis as a next step.

### Model‐based hierarchical drift diffusion model results

**FIGURE 4:**
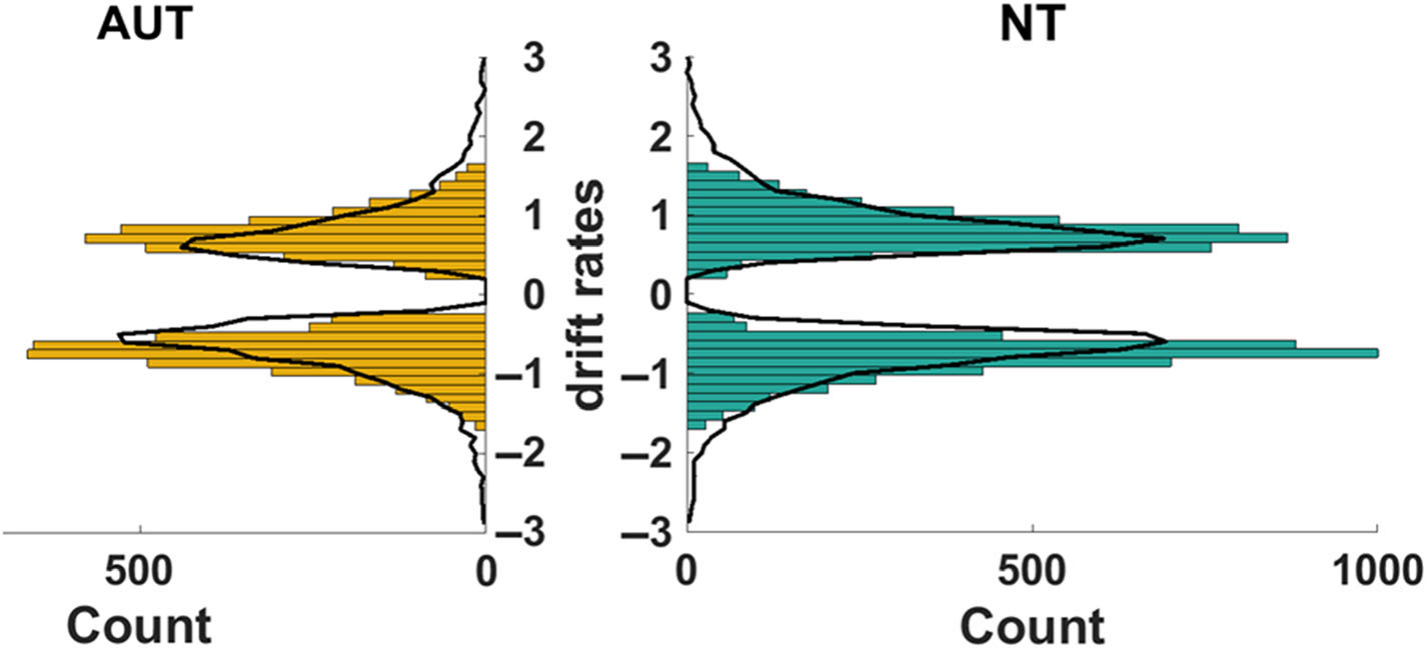
Posterior predictive checks (PPC) on the group level for the drift rates. Observed data is plotted as colored histogram and the simulated data is plotted as black line. For both groups, the simulated data appears to describe the observed data well. Positive drift rate values indicate “male” responses, negative ones “female.” PPC plots for both groups across all conditions can be found in Supporting Information: 7.

First, supporting our model's validity, the PPC showed good alignment of the observed data and reaction times yielded by simulations based on the hDDM parameters (Figure 4, Supporting Information: 7 for PPC plots separately for all conditions).

**FIGURE 5:**
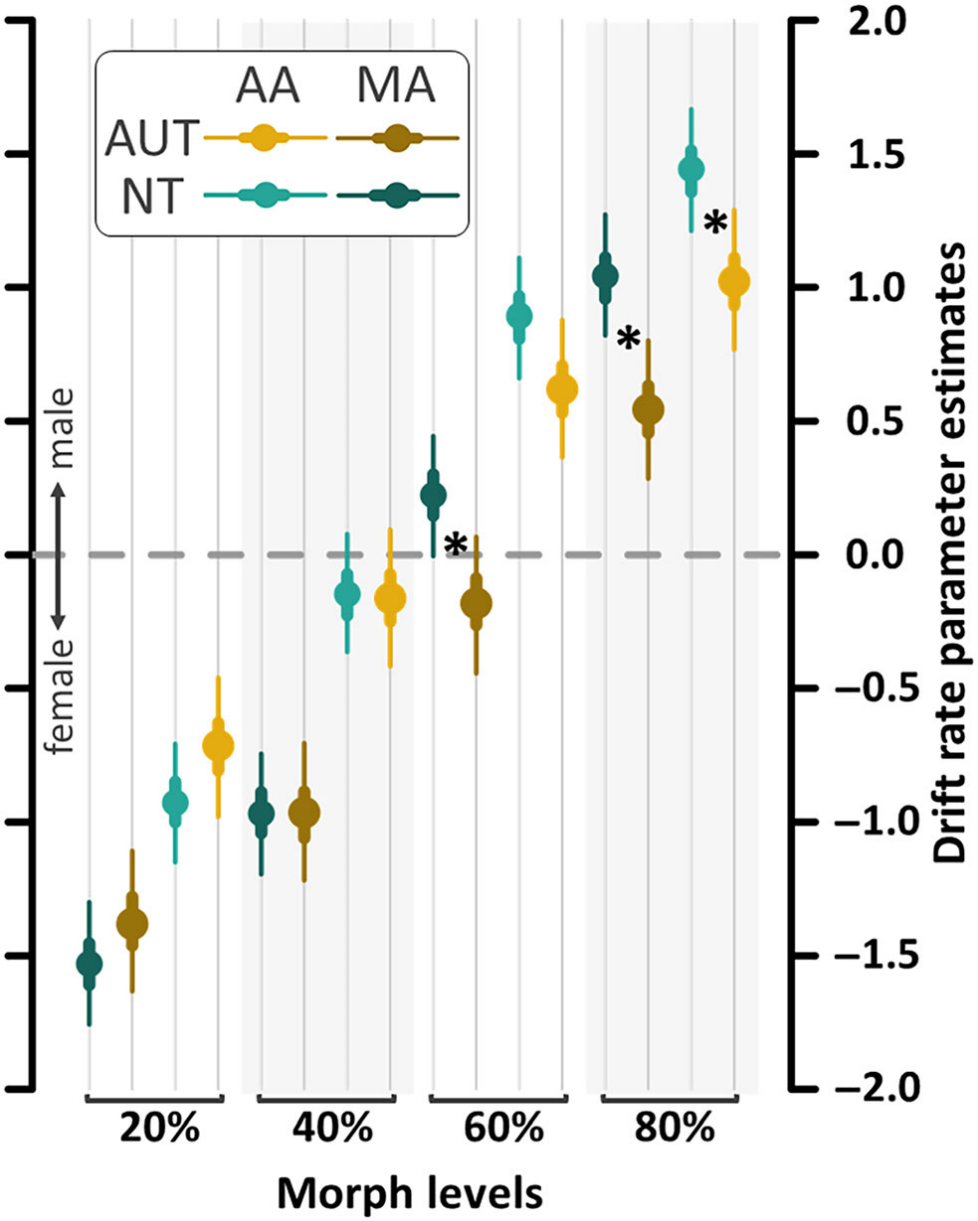
Drift rates for the autism (AUT) and the neurotypical (NT) group, estimated for each Morph level (20/40/60/80% male) and Adaptor (androgynous = AA and male = MA). Points represent the median, thick lines the 50% and thin lines the 95% credible interval of the parameter estimate. Expectedly, the Adaptor differences are larger in the 40% and 60% Morph levels, indicative of a successful face aftereffect in both groups. Asterisks indicate the three significant group differences. Note that, although not specifically indicated in the figure, every Adaptor difference (AA vs. MA) is significant.

Next, to check whether the FAE impacted the speed of the information accumulation process, we tested the drift rates across all Morph levels and both Adaptor conditions. These analyses revealed credible effects of Adaptor in both Groups and for all Morph levels (Figure 5; all Group and Adaptor comparison figures including the HDI statistics are in Supporting Information: 8 and 9). Expectedly, the FAE differences were significantly larger in the more ambiguous 40% and 60% Morph levels (HDI_97.3%_ = [0.004; 0.46]).

**FIGURE 6:**
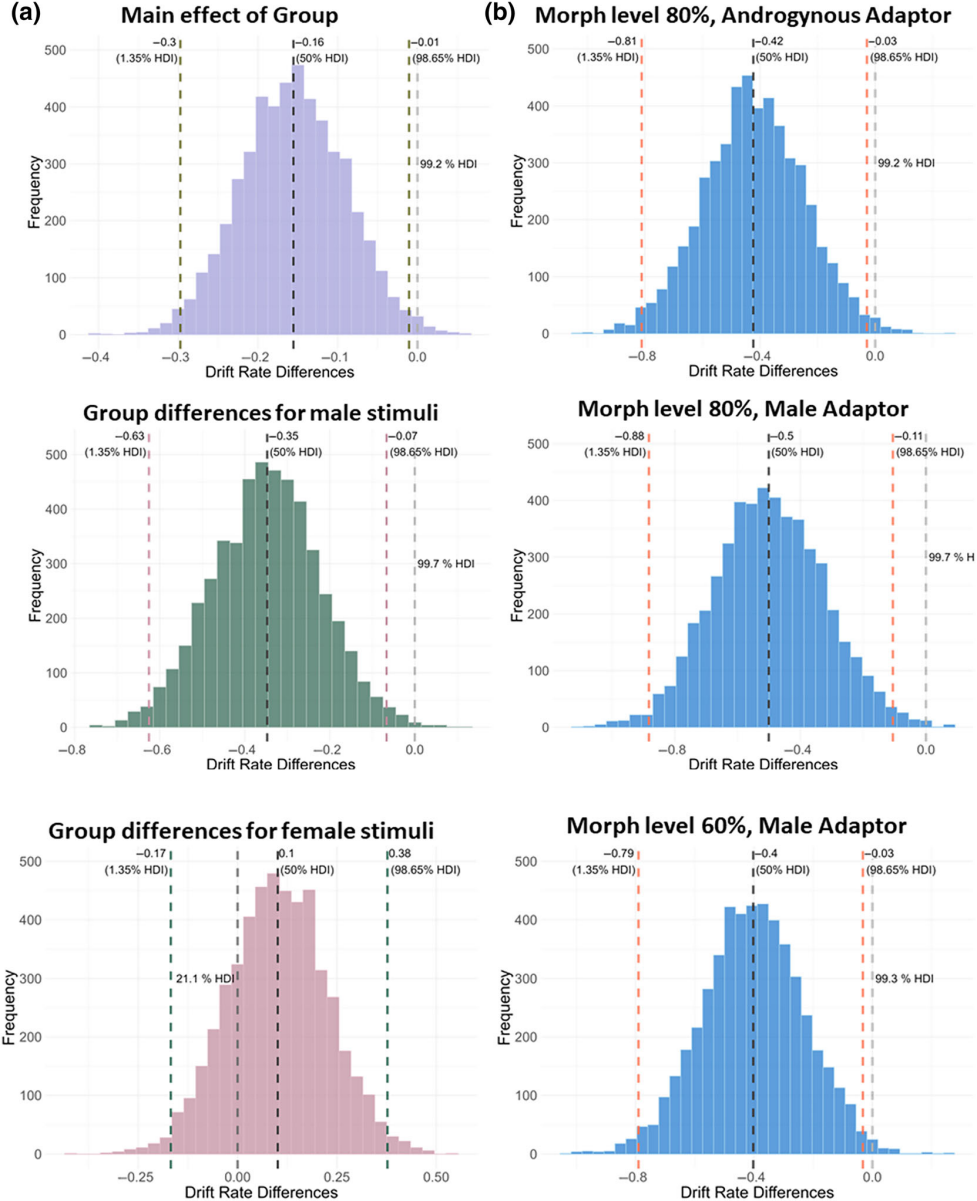
Histograms depicting the high density intervals (HDI) of the group differences in the drift rates (AUT‐NT). (a) HDIs of the drift rates for the main effect of group plus the group differences separately for male (60 and 80%) and female (20 and 40%) stimuli in the androgynous Adaptor condition. (b) HDIs of the significant group comparisons in the Group × Adaptor × Morph level hierarchical drift diffusion model derived drift rates. Negative values represent lower drift rates for the group with autism‐spectrum disorder compared to the neurotypical group. Colored dashed lines represent the 1.35th and the 98.65th percentile. The black dashed line represents the median at the 50th percentile. The gray dashed line is positioned at 0 and has to be outside the 97.3% HDI for the group comparison to be considered significant. AUT, group on the autism spectrum; NT, neurotypical group.

In order to probe our “sensory precision” hypothesis in autism, we first investigated whether the autistic group showed faster information accumulation towards the correct response than the NT group. Surprisingly, we found that overall, the autistic group exhibited a lower drift rate than the NT group (Figure 6a; HDI_97.3%_ = [−0.30; −0.01]), indicative of a slower information accumulation towards the correct response. Interestingly, at a closer inspection, this effect indicated an “own‐gender bias.” For the predominantly female stimuli (Morph level 20% and 40%), groups showed no significant group differences (HDI_97.3%_ = [−0.17; 0.38]). However, for the predominantly male stimuli (Morph level 60% and 80%), the group on the autism spectrum demonstrated lower drift rates compared to the NT group (HDI_97.3%_ = [−0.63; −0.07]), suggesting that the NT group exhibited an own‐gender bias while the autistic group did not. When comparing the 16 individual drift rates of the Group × Adaptor × Morph level interaction, the autistic group demonstrated lower drift rates in both Adaptor conditions in the 80% Morph level as well as in the male Adaptor gender condition in the 60% Morph level (Figure 6b; androgynous, 80%: HDI_97.3%_ = [−0.81; −0.03]; male, 80%: HDI_97.3%_ = [−0.88; −0.11]; male, 60%: HDI_97.3%_ = [−0.79; −0.03]) compared to the NT group. Together, these findings do not support the hypothesis that autism is characterized by increased sensory precision (reflected by higher drift rates) compared with NT controls.

**FIGURE 7:**
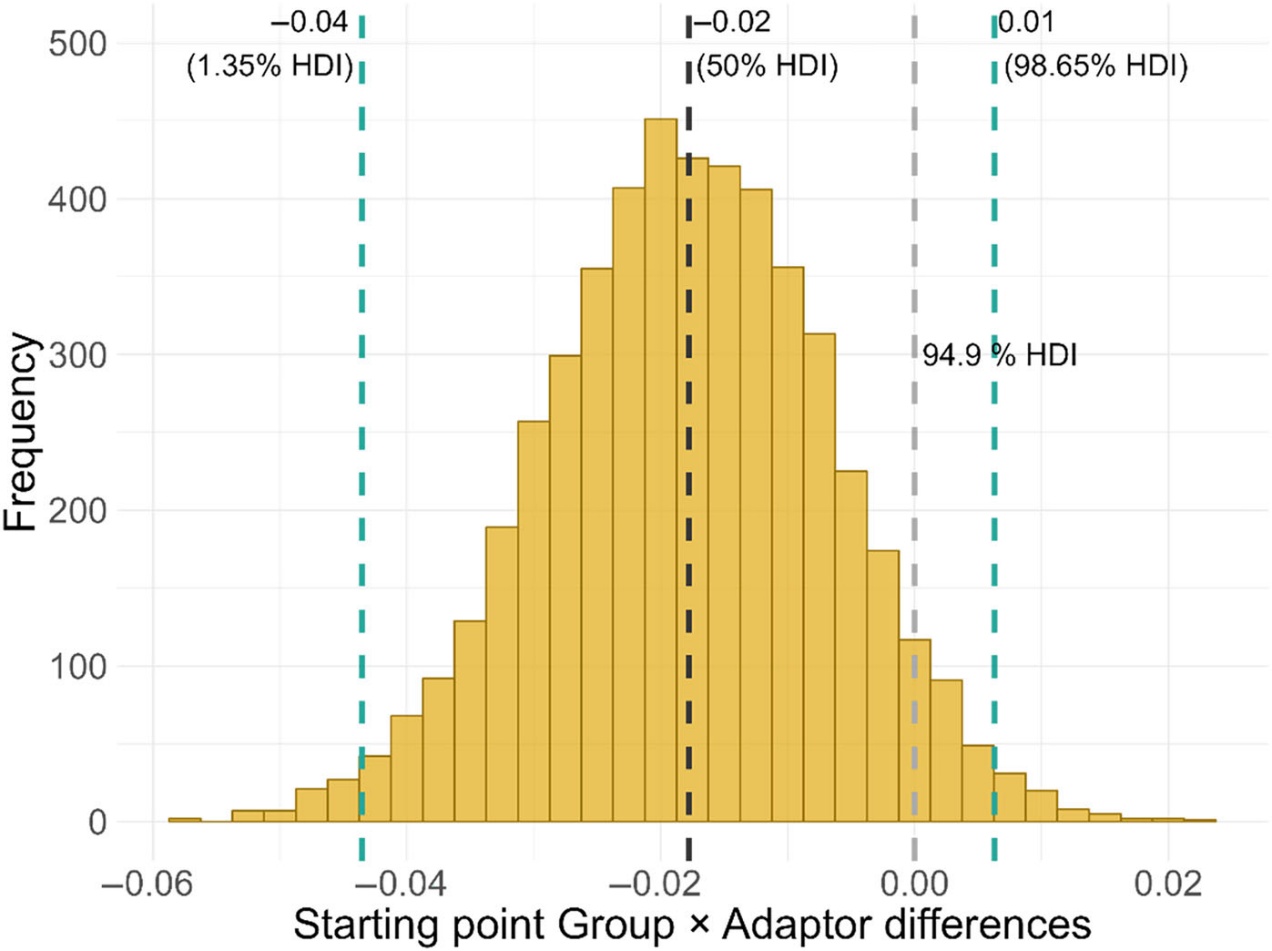
Histogram depicting the high density interval (HDI) of the nested differences of the Group × Adaptor interaction of the starting point parameters. Negative values indicate a weaker Adaptor effect on the starting point in the group with autism. Green dashed lines represent the 1.35th and the 98.65th percentile. The black dashed line represents the median at the 50th percentile. The gray dashed line is positioned at 0 and has to be outside the 97.3% HDI for the interaction to be considered significant.

Next, we tested the “imprecise priors” hypothesis, which should be reflected by differential group differences in the starting point parameter as well as the drift rates modulated by the FAE, with a less pronounced FAE for the autism group. However, we were not able to find any group‐specific effects of Adaptor on the starting point *b* (Figure 7, Supporting Information: 10). Additionally, both groups showed similar changes of the drift rates when challenged by the FAE (Supporting Information: 11; HDI_97.3%_ = [−0.04; 0.01]). Thus, the data provide no evidence for the “imprecise priors” hypothesis, instead indicating intact priors in autism.

Lastly, groups did not differ with respect to the boundary *a* (Supporting Information: 12; HDI_97.3%_ = [−0.17; 0.19]). However, the autistic group had a lower non‐decision time *t* compared to the NT group (Supporting Information: 13; HDI_97.3%_ = [−0.14; −0.03]).

### Exploratory correlation analyses

To investigate whether the reduced drift rate parameters in the autism group were associated with the degree of autistic traits, we computed exploratory Spearman correlations between the SRS values and the mean drift rates (overall and for the male test faces). However, there was no indication of an association (overall: *r* = −0.026, *p* = 0.907; male test faces: *r* = −0.052, *p* = 0.812). Similarly, given the group difference in depressive symptoms (DIKJ), we aimed to investigate whether the degree of slowing in the drift rates in the autistic group might be driven by these symptoms, but observed no evidence for such an association (overall: *r* = 0.107, *p* = 0.671; male test faces: *r* = 0.095, *p* = 0.706).

## DISCUSSION

The current study sought to investigate perceptual processes in adolescent autism using a model‐free (employing frequentist statistics) as well as a model‐based (Bayesian hDDM) analytical approach on data of a gender FAEs task. More specifically, drawing on previous research related to the “Bayesian brain” in autism (e.g. Pellicano & Burr, 2012) and reports of diminished FAE not related to gender (e.g. Ewing et al., 2013; Pellicano et al., 2007; Rhodes et al., 2014), we pursued two hypotheses aimed at explaining the perceptual challenges frequently described by individuals with autism. First, we explored the “sensory precision” hypothesis, expecting that the autistic group would exhibit an overall accelerated rate of information accumulation compared to NT individuals. Second, we examined the “imprecise priors” hypothesis, where we anticipated that individuals on the autism spectrum would demonstrate a less pronounced FAE on accuracy and information accumulation speed, as well as the decision‐making starting point (bias). We found no evidence for the “imprecise priors” hypothesis (no diminished FAE in the autism group) and, regarding the hypothesis of “sensory precision,” we observed an opposite effect that was potentially driven by an own‐gender bias (reduced drift rates).

While several studies have identified diminished aftereffects in young autistic samples across various perceptual domains (face identity: Webster & MacLeod, 2011; Ewing et al., 2013; Pellicano et al., 2007, gaze direction: Rhodes et al., 2014, facial expression: Pellicano et al., 2013), our study did not observe the hypothesized diminished gender FAE predicted by our “imprecise priors” hypothesis. This observation was consistent for both the accuracy data in our model‐free approach and the hDDM parameters drift rate and starting point in the model‐based approach. Concerning the question of qualitative (“how”) and quantitative (“how much”) perceptual differences in face processing in autism, our findings suggest that adolescents with autism do not exhibit qualitative differences in gender face adaptation, indicating the presence of intact priors. Similarly, intact priors have been inferred in individuals on the autism spectrum in other experiments, for example, when focusing on gaze direction (Palmer et al., 2018; Pell et al., 2016), when testing the “light from above” assumption (Croydon et al., 2017) or when performing a decision‐under‐uncertainty paradigm (Randeniya et al., 2021). Furthermore, recent work by (Ward et al., 2024) supports the notion of intact priors in autism, utilizing a gaze direction task alongside EEG measurements, thus providing neurophysiological evidence that complements our behavioral observations. Consequently, while previous work indicates that there is diminished norm‐based adaptation in autism possibly due to imprecise priors, particularly in face perception, our findings raise the possibility that imprecise priors do not extend to gender face processing. Notably, the gender FAE in our adolescent groups appeared stronger compared to the findings in the adult sample by (Kloth et al., 2010). This observation is in line with developmental research showing that aftereffects are stronger in children and adolescents and weaken with age (e.g. Cohen Kadosh et al., 2013, Hills et al., 2010). Overall, our results suggest that gender face processing may not be qualitatively altered in adolescents with autism, providing supporting evidence for the presence of intact priors.

Our findings related to the “sensory precision” hypothesis indicate an effect in the opposite direction, with autistic adolescents exhibiting decreased sensory precision irrespective of the FAE. More specifically, the autistic group showed generally lower drift rates, suggesting slower sensory information integration compared to the NT group. Considering previous work observing increased sensory precision in autism (Lawson et al., 2014; Lawson et al., 2015; Palmer et al., 2017; Van De Cruys et al., 2014) as well as in NT samples exhibiting autistic traits (Karvelis et al., 2018), this result is somewhat surprising. However, using a two‐alternative forced‐choice random dot motion task and similarly applying an hDDM model, Schneebeli and colleagues (2022) were also not able to confirm this hypothesis and also found evidence for lower drift rates in the autistic group. Interestingly, when comparing drift rates for the two test face genders, our data revealed a gender‐specific effect. In detail, we observed reduced drift rates only for male test faces in the individuals on the autism spectrum (which were all male), but not for female test faces. The significant (frequentist) Morph level × Group interaction lends even further support for this observation—with the autistic group exhibiting difficulties accurately identifying male test faces. This effect could be driven by the lack of the own‐gender bias in the drift rates of the individuals with autism. The own‐gender bias is a well‐documented phenomenon in NT populations, where individuals tend to recognize and process faces of their own gender more efficiently (e.g. Wolff et al., 2014). Although this phenomenon is traditionally stronger in females, males also typically demonstrate it, though with more variability (Herlitz & Lovén, 2013). Our results regarding the influence of the own‐gender bias—or the absence thereof in autism—on information accumulation speed resonate with prior research suggesting gender‐selective coding mechanisms during gender face processing (Jaquet & Rhodes, 2008; Little et al., 2005; Rhodes et al., 2011). Speculatively, these group differences in the drift rates for male stimuli could indicate that such biases are potentially modulated by individual differences and experiences, especially during early development (Webb et al., 2017a). Accordingly, our findings may be a result of reduced face memory capacities reported in autism (Weigelt et al., 2012), potentially slowing early development of own‐group related information processing (gender information) corresponding with the expert face processing hypothesis (Mares et al., 2024; Schwaninger et al., 2003). On a similar note, our findings may indicate a delayed development of the face space in autism (Strauss et al., 2012), leading to more subtle qualitative differences. Furthermore, in the context of the own‐gender bias, it has been shown that during the processing of own‐gender faces, eyes are fixated longer and more frequently (Man & Hills, 2016). In autism, however, face processing is presumably characterized by avoiding the eyes (Tanaka & Sung, 2016), which could diminish the advantage in processing own‐gender faces, thus resulting in a reduced speed of information accumulation leading to the absence of the own‐gender bias in autism.

### Limitations

When interpreting our findings, several limitations must be considered. First, there are limitations with respect to our sample and its composition: (1) our sample size was relatively small, possibly preventing us from observing smaller effects; (2) our study included only male individuals with autism (and only male NT), not allowing conclusions about women on the autism spectrum; (3) we only included individuals without intellectual disabilities, making it uncertain whether our results generalize to the broader spectrum of autism; (4) our focus on adolescent participants precludes conclusions about these mechanisms during earlier and later developmental stages. Second, the FAE task we employed consisted only of male and androgynous adaptors, omitting female adaptors. Similarly, our reliance on binary gender categorization in the task may not fully capture the complexities of gender perception in the face space (Cronin et al., 2017). Third, the two groups differed with respect to their depressive symptom scores. While there was no correlation between the depression score and the drift rate difference in the autistic group, there remains a possibility that—at least partially—the results are driven by this difference in symptoms. Lastly, our experiment did not impose a time limit, which in itself can diminish aftereffects; however, we observed strong aftereffects in both groups.

### Conclusion

Taken together and building on the “Bayesian brain” framework, our study found no evidence for sensory dominance in adolescents with autism using a FAEs task, contrary to the hypotheses of increased sensory precision or imprecise priors. Instead, we observed indications for decreased sensory precision, suggesting a potential absence of the own‐gender bias in face processing among individuals on the autism spectrum. These findings call for a more nuanced understanding of perceptual processing in this population. Specifically, while previous studies have documented diminished FAE in other domains in autism, our results highlight that gender face adaptation may not be universally affected by the perceptual challenges commonly associated with autistic traits.

Future research should continue to employ model‐based approaches, particularly within the “Bayesian brain” framework, to further explore the nuanced and complex perceptual alterations in autism. Pursuing these lines of research could help to gain a deeper understanding of the “Bayesian brain” in neurodiverse populations and consequently inform the development of more targeted interventions.

## CONFLICT OF INTEREST STATEMENT

VR has received payment for consulting and writing activities from Lilly, Novartis, and Shire Pharmaceuticals, lecture honoraria from Lilly, Novartis, Shire Pharmaceuticals, and Medice Pharma, and support for research from Shire and Novartis. He has carried out (and is currently carrying out) clinical trials in cooperation with the Novartis, Shire, and Otsuka companies.

## ETHICS STATEMENT

The study was approved by the Institutional Review Board of Technische Universität Dresden (Ethikkommission an der Technischen Universität Dresden), Germany, and carried out in accordance with the latest version of the Declaration of Helsinki, and all participants (and their guardians if underage) gave written informed consent.

## Supporting information


**Data S1:** Supporting Information.

## Data Availability

The datasets used and/or analyzed during the current study are available from the corresponding author on reasonable request.
