# Supplementary material for: Disentangling the perceptual underpinnings of autism: Evidence from a face aftereffects experiment
Source: Autism Res. 2024 Dec 16;18(2):349–61. doi: 10.1002/aur.3283 (PMC11826018; doi:10.1002/aur.3283)
Supplement: Supplementary file 1 — Data S1: Supporting Information. [file AUR-18-349-s001.docx]

**Supplementary Materials**

Disentangling the Perceptual Underpinnings of Autism:

Evidence from a Face Aftereffects Experiment

Julius Hennig, Arne Doose, Clara Marie Breier, Alexander Soutschek, Nicole Beyer, Stefan Schweinberger, Ingeborg Kamp-Becker, Luise Poustka, Katja Albertowski, Veit Roessner, Stefan Ehrlich

Contents

[1. Illustrative example of sensory dominance during perception 2](#_Toc164150161)

[2. Sample selection and exclusion criteria 3](#_Toc164150162)

[2. Diagnostical procedures 4](#_Toc164150163)

[3. Hierarchical Drift Diffusion Model (hDDM) Diagnostics – Trace and Density plots 6](#_Toc164150164)

[4. Posterior Predictive Checks (PPC) 8](#_Toc164150165)

[5. hDDM Results – Group effects in drift rates 10](#_Toc164150166)

[6. hDDM Results – Face Aftereffect in drift rates 11](#_Toc164150167)

[7. hDDM Results – Starting point 12](#_Toc164150168)

[8. hDDM Results – Interaction of Group and Adaptor 13](#_Toc164150169)

[9. hDDM Results – Boundaries 14](#_Toc164150170)

[10. hDDM Results – Non-decision time 15](#_Toc164150171)

[References 16](#_Toc164150172)

# 1. Illustrative examples of shifted precision weights during perception

**Example 1 – redecorated living room:** Imagine entering a friend’s apartment, unaware that they have just redecorated their living room. Several outcomes are possible depending on how you process information. If you rely on well-balanced priors and sensory input (with respect to their precision weights), with your friend next to you, you would know you are in their living room based on your internal map of the apartment. And, while a lot of furniture has been replaced, you could recognize the old sofa, now on the opposite wall. Upon processing all the changes you notice (prediction errors), you update your “belief”, your internal image, of your friend’s living room. However, if you have imprecise priors and/or show increased sensory precision, you might struggle to integrate these changes with your previous memory of this room as you focus excessively on each new detail without recognizing the overall familiarity of the setting. This could lead you to perceive the room as being “out of place”, resulting in the creation of a completely new internal image of your friend’s living room, rather than just slightly updating the existing one (overfitting and lack of generalization).

**Example 2 – birdwatching:** Imagine you are birdwatching in a familiar forest, one you have visited many times before. You have a good mental map of which bird species are usually found in different parts of this forest. If your priors are well-calibrated and you balance them with the sensory input, you easily recognize the usual suspects by their colors and songs, even if there are some rare visitors among them. Your prior knowledge helps you quickly categorize and identify these birds accurately. However, if your priors are imprecise and/or you rely too heavily on detailed sensory input, you might struggle to recognize even common birds. Each slight variation in color, song or in which tree they sit might lead you to question your recognition, possibly concluding that these are entirely different species than those you know. This intense focus on sensory details could make the habitat feel unfamiliar and confusing, as you can't easily match what you see and hear with what you expect based on your previous visits.

# 2. Sample selection, exclusion criteria and additional sample information

Further exclusion criteria: Traumatic brain lesions; psychotic disorder, obsessive-compulsive disorder, social anxiety disorder, major depressive episode with suicidal ideation, aggressive behavior, any personality disorder; serious neurological disease (e.g. epilepsy), anamnestic known metabolic or endocrinological disorders; cardiac disorders.

With respect to the absence of ASD symptomatology in the NT group, we applied the following rules using the Autism Quotient (AQ) and Social Responsiveness Scale (SRS) scores:

1. For the AQ, we included all participants using a threshold of 29 as suggested by Broadbent and colleagues (1) (n=39/39)
2. For the SRS, we only included all NT participants:
   1. showing “high social reactivity” (T values <40; n=19/39)
   2. showing “normal reactivity” (T values between 40-60; n=18/39)
   3. showing “low or mid impairments in social reactivity” (T values between 61-75; n=2/39 with T values of 61 and 63) only if the passed the stricter AQ criterion of <20 (n=2/39)

Regarding psychotropic medication, n=6 participants with autism reported stable intake of one or more drugs: n=5 with stimulants (e.g. Ritalin), n=1 atypical neuroleptics and n=1 typical neuroleptics. N=3 individuals in the autism group were diagnosed with comorbid disorders: n=1 with Attention-deficit hyperactivity disorder (F90.0), n=1 with attention deficit disorder without hyperactivity (F98.8) and n=1 with combined vocal and multiple motor tic disorder (F95.2).

# 3. Diagnostical procedures

**ADOS.** The ADOS is a semi-structured, standardized assessment that involves direct observation of an individual's social interaction, communication, and play behaviors (2). The ADOS consists of a series of activities and tasks that are designed to elicit social and communication behaviors that are commonly impaired in individuals with autism.

**ADI-R.** The ADI-R is a structured, standardized interview that is administered to a caregiver or family member of the individual being evaluated for ASD (3). The ADI-R assesses the individual's social and communication behaviors, as well as their repetitive and restricted interests and behaviors.

**SRS.** The Social Responsiveness Scale (SRS) is a quantitative self-report measure designed to assess social competence and social communication skills in individuals on the autism spectrum and related developmental disorders (4). The SRS is typically completed by a caregiver or teacher who knows the individual well, and includes items that measure social behavior, reciprocal social communication, social awareness, social cognition, and social motivation. The SRS has been shown to have good reliability and validity for measuring social responsiveness in individuals with autism, and is commonly used in both clinical and research settings (5).

**AQ.** The Autism Quotient (AQ) is a self-report questionnaire designed to measure traits associated with autism in individuals without a clinical diagnosis of ASD (6). The AQ consists of 50 items that assess social skills, attention switching, communication, imagination and attention to detail. Respondents indicate the extent to which they agree or disagree with each statement on a 4-point Likert scale. Values below 26 can be considered as not showing any clinically significant levels of autistic traits (7).

**IQ.** IQ in the autistic sample was assessed with the complete version of the German adaptation of the Wechsler Adult Intelligence Scale (8). For the NT sample, we used a short version to rule out an IQ <75.

**MINI-KID.** The M.I.N.I.-KID (Mini International Neuropsychiatric Interview for Children and Adolescents) is a structured diagnostic interview designed to assess psychiatric disorders in children and adolescents (9). The interview covers a range of psychiatric disorders, including mood disorders, anxiety disorders, behavior disorders, and substance use disorders, among others. Its aim is to provide a reliable, valid, and efficient method for diagnosing psychiatric conditions in the pediatric population.

**SCL-90-R.** For general psychopathology, we used the self-report instrument Symptom Checklist-90-R (10). The symptomatology based on the Global Severity Index was considered significant for any participant with a mean value >63 which was the case for n=0.

**DIKJ.** The "Depressions-Inventar für Kinder und Jugendliche" (DIKJ; Depression Inventory for Children and Adolescents) is a German-language self-report questionnaire designed to assess symptoms of depression in children and adolescents (11). The DIKJ has been shown to have good reliability and validity for measuring depression in children and adolescents (12).

# 4. Hierarchical Drift Diffusion Model (hDDM) Diagnostics – Trace and Density plots

The trace plots of the Markov Chain Monte Carlo (MCMC) simulations indicate good convergence for all 2 × 2 × 4 drift rates during the 5,000 remaining iterations (after the 15,000 iterations of the burn-in phase) by showing a stationary distribution around an equilibrium. Accordingly, the density plots support this conclusion by showing normal distributions for the drift rates. Additionally, the $\hat{R}=1$ shows an excellent convergence between the two chains for all drift rates.


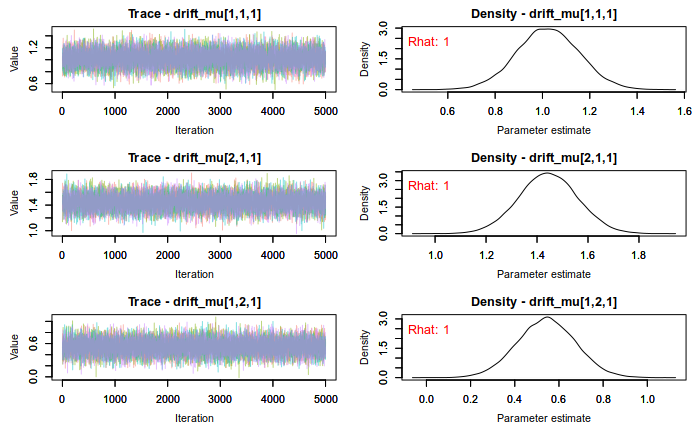


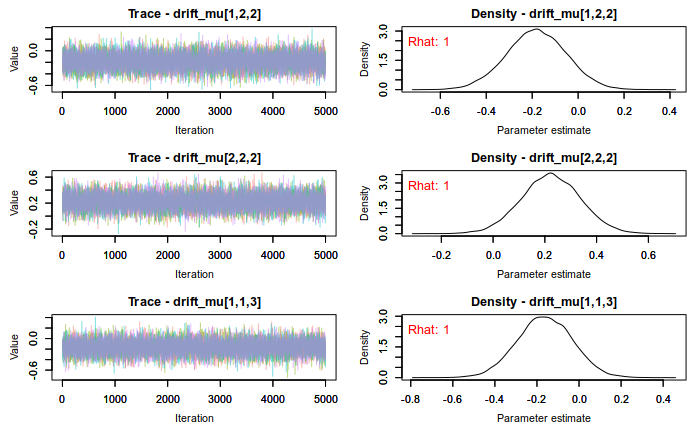


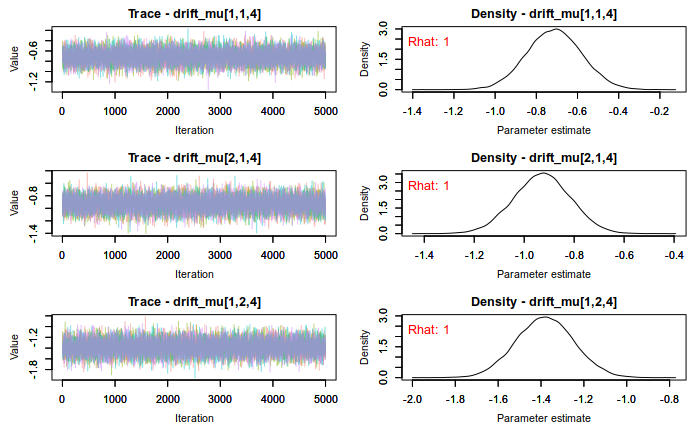


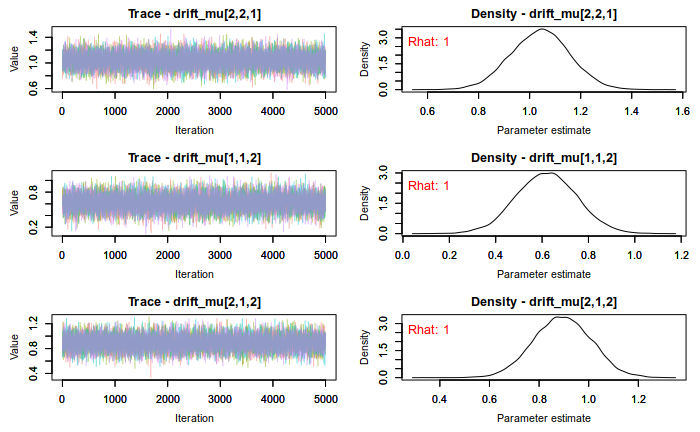


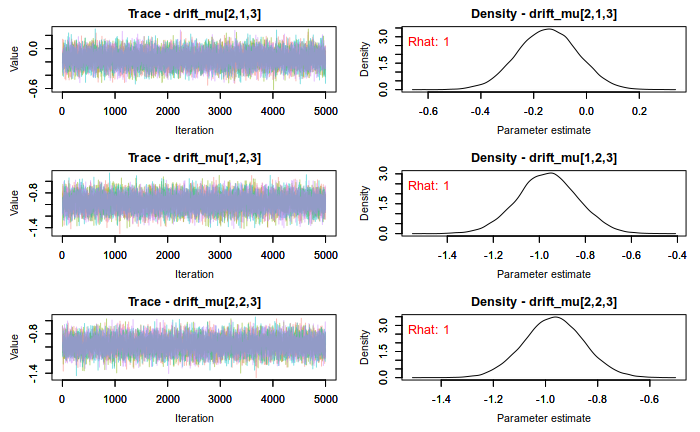


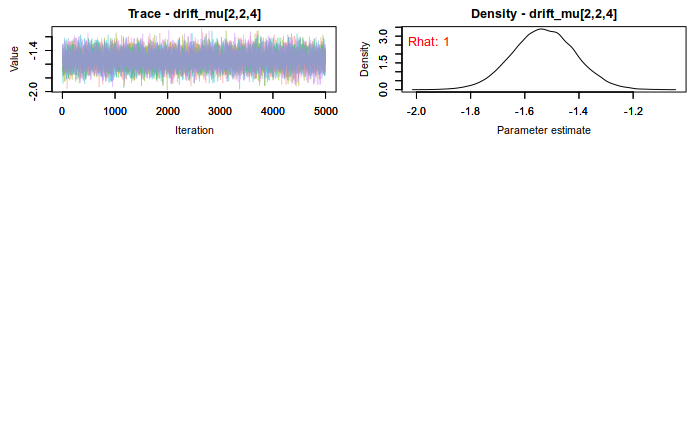


**Figure S1.** Trace and density plots for all 16 drift rate parameter estimates. The numbers in the brackets indicate group [1=AUT, 2=NT], adaptor [1=androgynous, 2=male] and morph level [1=80% male, 2=60%, 3=40%, 4=20%] in this order. $\hat{R}=1$ represents optimal convergence between the two Markov Chain Monte Carlo chains. AUT=individuals on the autism spectrum; NT=neurotypical individuals.

# 5. Example of nested subtraction of hDDM parameters


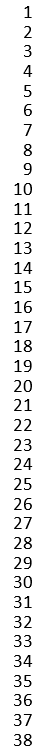
The following R code snippet illustrates how we calculated the nested subtractions based on the approach suggested by Johnson et al. (13). Here, we use the example of the Group × Adaptor interaction for each of the four Morph levels for the drift rate parameters. As described in the Methods section, this procedure calculates subtractions for sets of 5,000 hDDM iterations (line 19-20). The data stored in the drift_cut array variable is structured as follows: drift_cut**[**iteration, group, adaptor, morph level**]** and contains 5,000 × 2 × 2 × 4 data points.

# 6. Model-free analysis – Addendum

In addition to the repeated-measures ANOVA reported in the main manuscript, we performed post-hoc t-tests for significant interactions of Group × Morph level and Adaptor × Morph level.

| **Table S1.** | | | | |
| --- | --- | --- | --- | --- |
| Results of the post-hoc t-tests for the significant interactions of the repeated-measure ANOVA of the face aftereffects (Bonferroni corrected). | | | | |
| Interaction | Morph level^a^ | Comparison | Mean Difference | *p* |
| Group × Morph level | 20% | AUT - NT | 0.025 | .530 |
|  | 40% | AUT - NT | -0.021 | .591 |
|  | 60% | AUT - NT | -0.094 | .025 |
|  | 80% | AUT - NT | -0.110 | .015 |
| Adaptor × Morph level | 20% | AA - MA | 0.125 | <.001 |
|  | 40% | AA - MA | 0.179 | <.001 |
|  | 60% | AA - MA | 0.176 | <.001 |
|  | 80% | AA - MA | 0.080 | <.001 |
| *Note.* ^a^ = Morph level represent the proportion of the male compared to the female stimulus; AUT = group on the autism spectrum; NT = neurotypical individuals; AA = androgynous adaptor; MA = male adaptor. | | | | |

To further explore the effects observed in our study, we conducted a Bayesian repeated measures ANOVA (rm-ANOVA) using JASP (14). This approach allows for the quantification of evidence in favor of or against specific hypotheses and it confirmed the results of our frequentist rm-ANOVA.

| **Table S2.** |  |  |  |
| --- | --- | --- | --- |
| Results of the Bayesian Repeated Measures ANOVA. | | | |
| Effects | P(incl\|data) | BF_incl_ | interpretation^a^ |
| Group | 0.876 | 2.53 | anecdotal |
| Adaptor | >0.999 | 5.74×10^13^ | extreme |
| Morph level | >0.999 | 5.74×10^13^ | extreme |
| Group × Adaptor | 0.218 | 0.60 | anecdotal |
| Group × Morph level | 0.738 | 6.10 | moderate |
| Adaptor × Morph level | 0.999 | 2491.50 | extreme |
| Group × Adaptor × Morph level | 0.013 | 0.23 | moderate |
| *Note.* P(inclldata) = posterior probability that a given effect is included in the model after considering the data; BF_incl_ = Bayes Factor, indicating how much more likely it is that the effect should be included in the model compared to being excluded, given the data (values >1 describe the degree of evidence for the alternative hypothesis, <1 for the null hypothesis); ^a^ = Bayes factor evidence levels according to Wagenmakers et al. (15). | | | |

# 7. Posterior Predictive Checks (PPC)

Here, we present a series of PPC plots. These plots compare the distributions of observed data with the distributions generated from our hierarchical drift diffusion model under repeated simulations. The close alignment between the simulated and observed data in these plots indicates that our model adequately captures the underlying patterns and variability in the experimental data, thus supporting the model's validity.


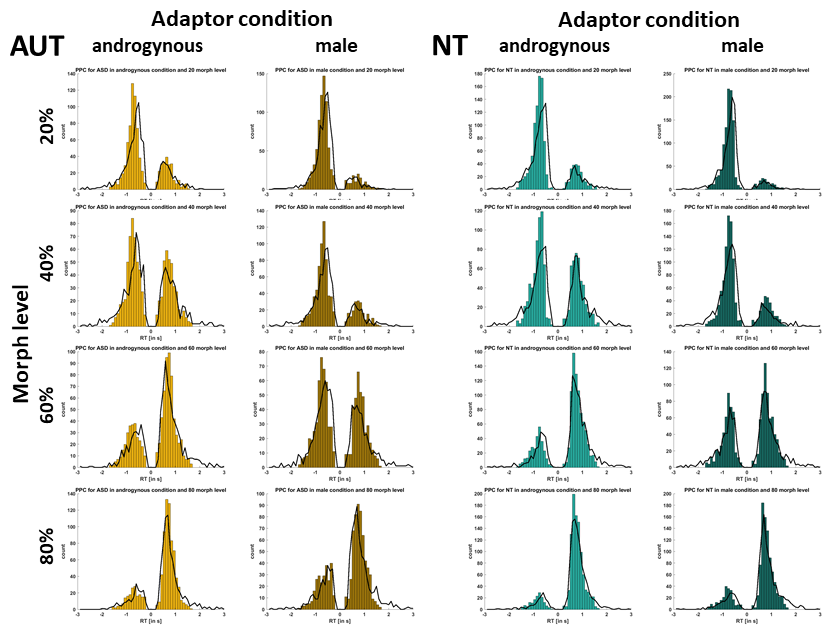


**Figure S2.** Posterior predictive checks (PPC) for the drift rates for both Groups, both Adaptor conditions and all four Morph levels. Observed data is plotted as colored histograms and the simulated data is plotted as a black line. AUT=individuals on the autism spectrum; NT=neurotypical individuals.

# 8. hDDM Results – Group effects in drift rates


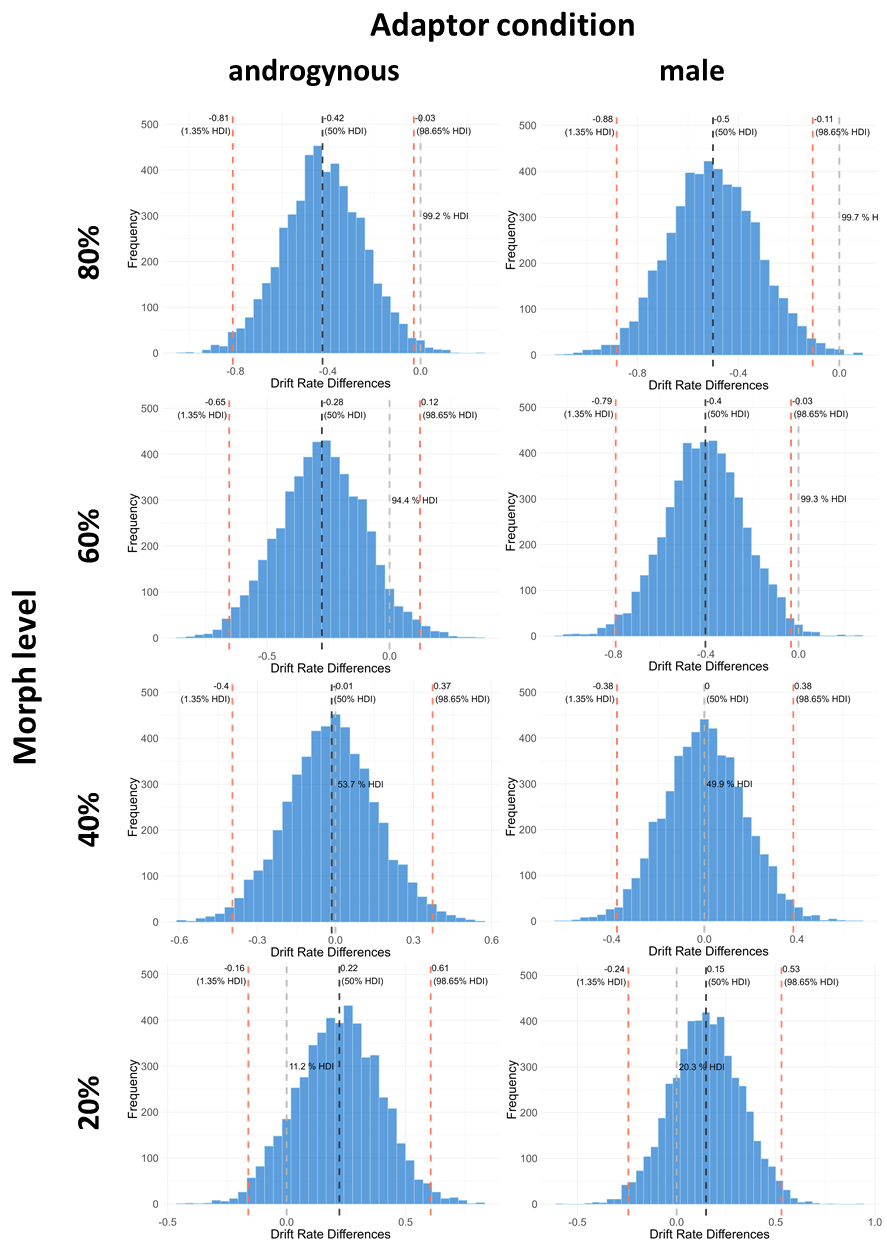


**Figure S3.** High density intervals (HDI) of the drift rates of all the Group comparisons for each Adaptor, with negative values representing lower drift rates for the group with autism-sprectrum disorder compared to the neurotyical group. Red dashed lines represent the 1.35^th^ and the 98.65^th^ percentile. The black dashed line represents the median at the 50^th^ percentile. The grey dashed line is positioned at 0 and has to be outside the 97.3% HDI for the group comparison to be considered significant.

# 9. hDDM Results – Face Aftereffect in drift rates


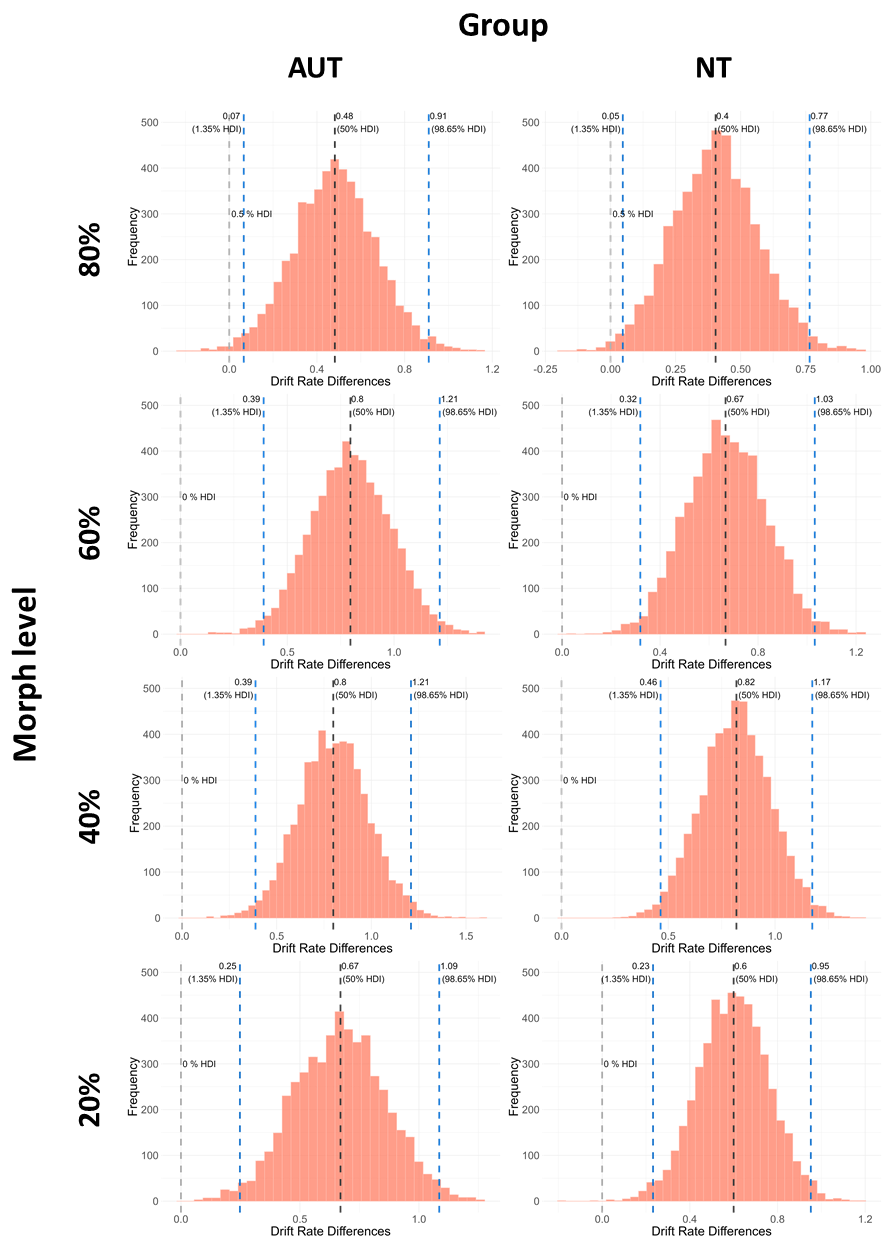


**Figure S4.** High density intervals (HDI) of the drift rates of all the Adaptor comparisons for each Group. Positive values represent the face aftereffect (androgynous–male). Blue dashed lines represent the 1.35^th^ and the 98.65^th^ percentile. The black dashed line represents the median at the 50^th^ percentile. The grey dashed line is positioned at 0 and has to be outside the 97.3% HDI for the group comparison to be considered significant, which is the case in all comparisons. ASD=group with autism-sprectrum disorder, NT=neurotyical group.

# 10. hDDM Results – Starting point


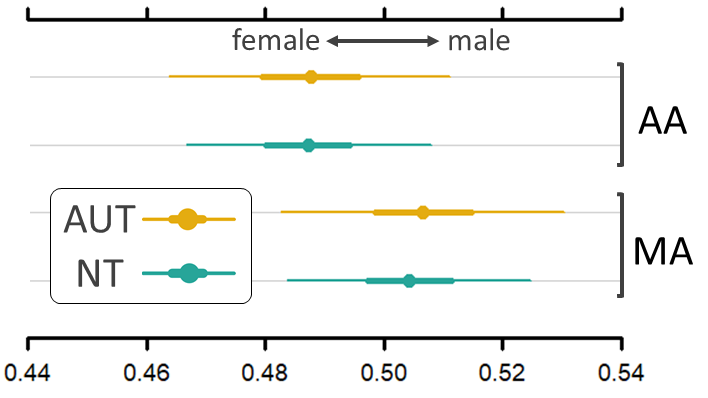


**Figure S5.** Starting point parameter for the autistic (AUT) and the neurotypical (NT) group, divided for adaptor gender (androgynous=AA and male=MA adaptor). Points represent the median, thick lines the 50% and thin lines the 95% credible interval of the parameter estimate.

# 11. hDDM Results – Interaction of Group and Adaptor


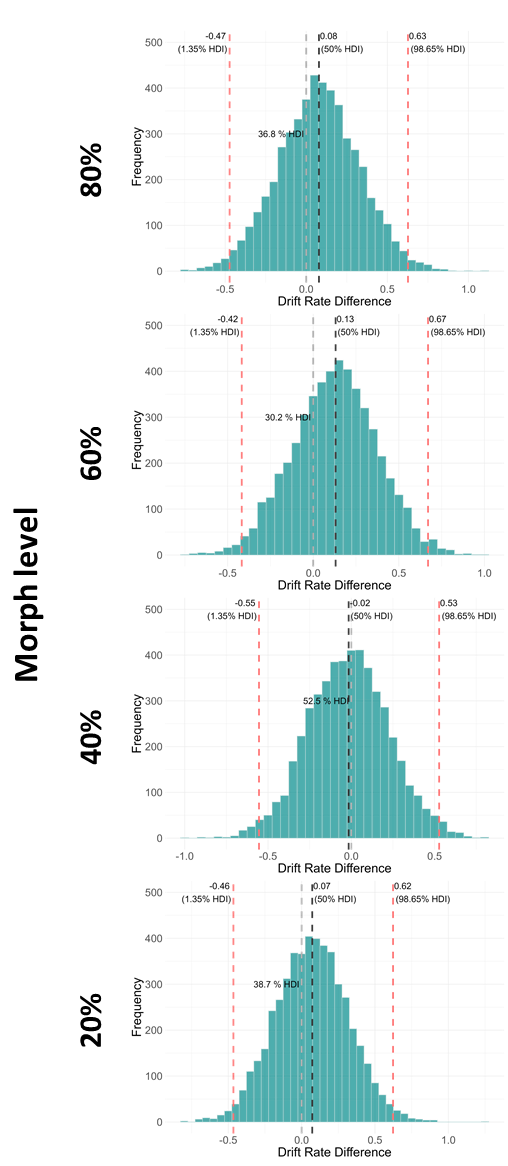


**Figure S6.** High density intervals (HDI) of the drift rates for the Adaptor x Group interaction for each Morph level. Negative values indicate a larger face aftereffect in neurotypical participants. Red dashed lines represent the 1.35^th^ and the 98.65^th^ percentile. The black dashed line represents the median at the 50^th^ percentile. The grey dashed line is positioned at 0 and has to be outside the 97.3% HDI for the group comparison to be considered significant.

# 12. hDDM Results – Boundaries


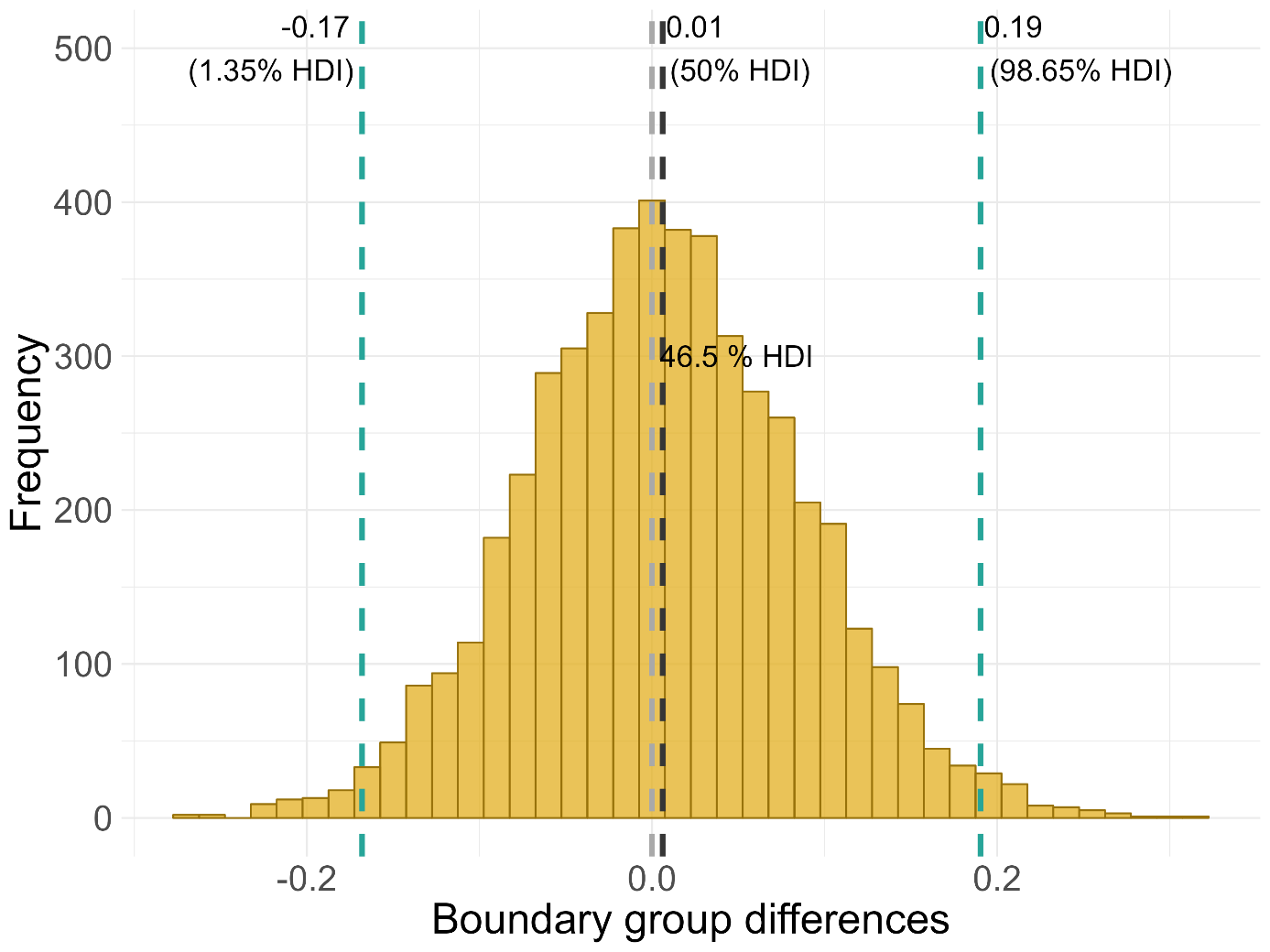


**Figure S7.** High density interval (HDI) of the boundary group differences. Green dashed lines represent the 1.35^th^ and the 98.65^th^ percentile. The black dashed line represents the median at the 50^th^ percentile. The grey dashed line is positioned at 0 and has to be outside the 97.3% HDI for the group comparison to be considered significant.

# 13. hDDM Results – Non-decision time


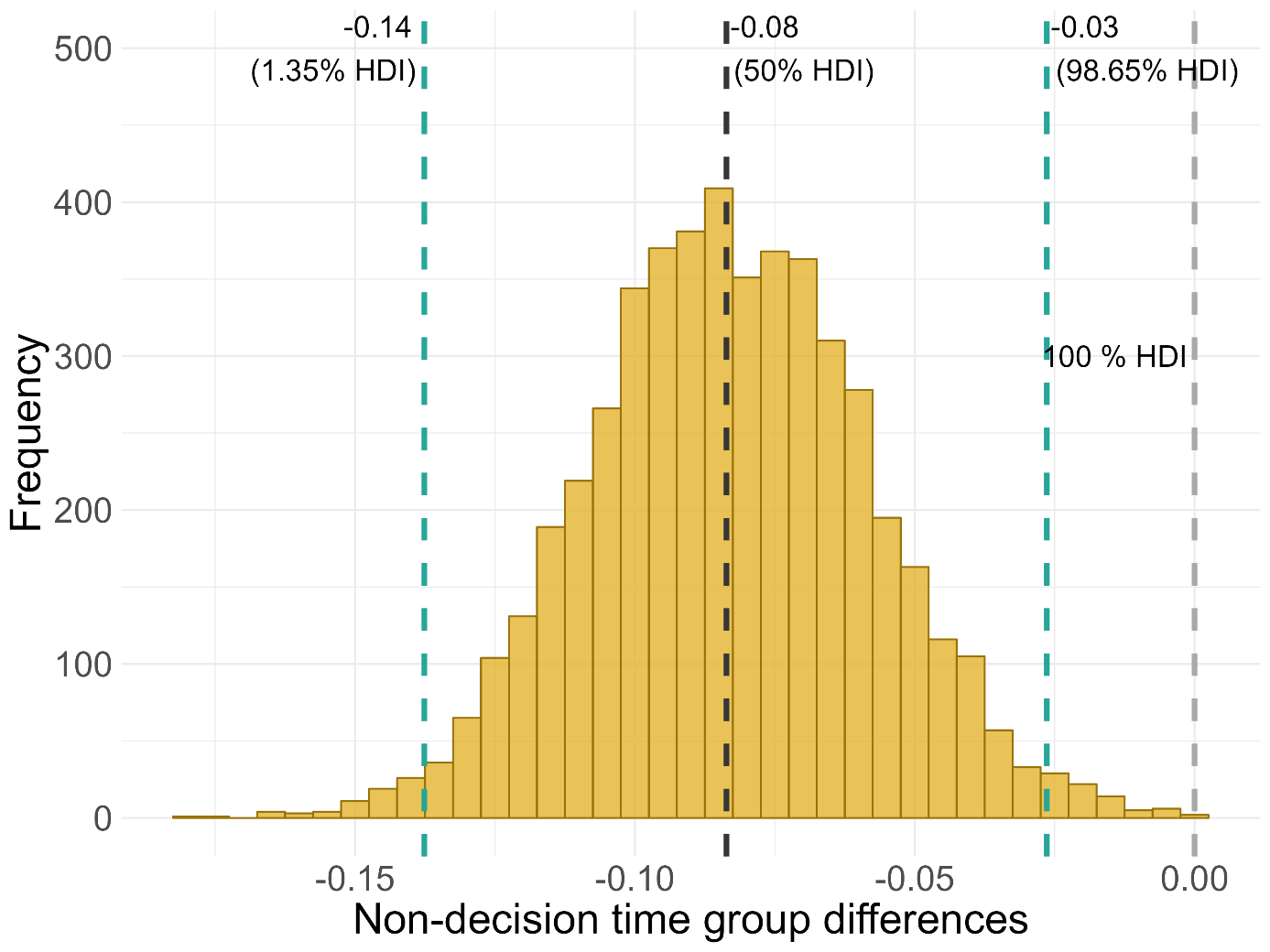


**Figure S8.** High density interval (HDI) of the non-decision time group differences. Negative values indicate a shorter non-decision time for the group on the autism spectrum compared to the neurotypical group. Green dashed lines represent the 1.35^th^ and the 98.65^th^ percentile. The black dashed line represents the median at the 50^th^ percentile. The grey dashed line is positioned at 0 and has to be outside the 97.3% HDI for the group comparison to be considered significant.

# References

1. Broadbent J, Galic I, Stokes MA. Validation of Autism Spectrum Quotient Adult Version in an Australian Sample. Autism Res Treat. 2013;2013:1–7.

2. Poustka L, Rühl D, Feineis-Matthews S, Poustka F, Hartung M, Bölte S. Beobachtungsskala für Autistische Störungen-2 (ADOS-2). Deutschsprachige Fassung der Autism Diagnostic Observation Schedule, Second Edition (ADOS-2) von C. Lord, M. Rutter, PC Di-Lavore, S. Risi, K. Gotham und SL Bishop (Teil I: Module 1 bis 4) und C. Lord, RJ Luyster, K. Gotham und W. Guthrie (Teil II: Kleinkind-Modul). Göttingen: Hogrefe; 2015.

3. Bölte S, Poustka F, Rühl D, Schmötzer G. Diagnostisches Interview für Autismus - Revidiert (Deutsche Fassung des Autism Diagnostic Interview - Revised von Michael Rutter, Ann Le Couteur und Catherine Lord). Göttingen: Hogrefe; 2006.

4. Constantino JN, Davis SA, Todd RD, Schindler MK, Gross MM, Brophy SL, et al. Validation of a Brief Quantitative Measure of Autistic Traits: Comparison of the Social Responsiveness Scale with the Autism Diagnostic Interview-Revised. J Autism Dev Disord. 2003;33(4):427–33.

5. Bölte S. Brief Report: The Social Responsiveness Scale for Adults (SRS-A): Initial Results in a German Cohort. J Autism Dev Disord. 2012 Sep;42(9):1998–9.

6. Baron-Cohen S, Hoekstra RA, Knickmeyer R, Wheelwright S. The Autism-Spectrum Quotient (AQ)—Adolescent Version. J Autism Dev Disord. 2006 Apr;36(3):343–50.

7. Woodbury-Smith MR, Robinson J, Wheelwright S, Baron-Cohen S. Screening Adults for Asperger Syndrome Using the AQ: A Preliminary Study of its Diagnostic Validity in Clinical Practice. J Autism Dev Disord. 2005 Jun;35(3):331–5.

8. von Aster MG, Neubauer AC, Horn R. WIE - Wechsler Intelligenztest für Erwachsene. Bern: Huber; 2006.

9. Lecrubier Y, Weiller E, Hergueta T, Allgulander C, Kadri N, Baldwin D, et al. M.I.N.I. – MINI INTERNATIONALES NEUROPSYCHIATRISCHES INTERVIEW, German Translation Version 6.0.0. 2010.

10. Franke GH, Derogatis L. SCL-90-R. Symptom-Checkliste von L. R. Derogatis - Deutsche Version. Göttingen: Beltz Test GmbH; 2002.

11. Stiensmeier-Pelster J, Schürmann M, Duda K. Depressions-Inventar für Kinder und Jugendliche (DIKJ). Göttingen: Hogrefe; 2000.

12. Keller F. Erfassung von Depressivität bei Kindern und Jugendlichen. Z Für Klin Psychol Psychother. 2016 Apr;45(2):148–50.

13. Johnson DJ, Hopwood CJ, Cesario J, Pleskac TJ. Advancing Research on Cognitive Processes in Social and Personality Psychology: A Hierarchical Drift Diffusion Model Primer. Soc Psychol Personal Sci. 2017 May;8(4):413–23.

14. JASP Team. JASP (version 0.19.0). 2024.

15. Wagenmakers EJ, Love J, Marsman M, Jamil T, Ly A, Verhagen J, et al. Bayesian inference for psychology. Part II: Example applications with JASP. Psychon Bull Rev. 2018 Feb;25(1):58–76.
